# Exploring the Therapeutic Effects of Psychedelics Administered to Military Veterans in Naturalistic Retreat Settings

**DOI:** 10.1002/brb3.70660

**Published:** 2025-07-07

**Authors:** Megan Calnan, Grace Blest‐Hopley, Chris Busch, Milly Adams, Simon G. D. Ruffell, Theodore Piper, Leor Roseman, Hannes Kettner, Robin Carhart‐Harris

**Affiliations:** ^1^ Centre for Clinical Brain Sciences, Division of Psychiatry University of Edinburgh Edinburgh UK; ^2^ King's College London, Institute of Psychiatry Psychology and Neuroscience London UK; ^3^ Health Analytics Collaborative Maryland USA; ^4^ Department of Medicine, Centre for Psychedelic Research Imperial College London London UK; ^5^ Onaya Science Iqiuitos Peru; ^6^ Social, Therapeutic and Community Studies and the Faiths & Civil Society Unit, Goldsmith University of London London UK; ^7^ School of Population and Global Health University of Melbourne Melbourne Australia; ^8^ Birmingham Law School University of Birmingham Birmingham UK; ^9^ Department of Psychology University of Exeter Exeter UK; ^10^ Psychedelics Division, Neuroscape University of California San Francisco USA

**Keywords:** ayahuasca, military, psilocybin, psychedelics, PTSD, retreats, veterans

## Abstract

**Background**: Military veterans are at risk of various mental health conditions, with profound implications for post‐deployment quality of life. Current treatment options encounter high dropout rates and non‐responsiveness, and overlook the importance of community building in veterans’ holistic recovery. Preliminary research suggests psychedelics offer therapeutic benefits for depression and post‐traumatic stress disorder (PTSD) in veterans. Integrating psychedelic therapies with a communal retreat setting could provide a more holistic framework for improving veterans’ well‐being.

**Objectives**: To evaluate the effects of psychedelic retreats on mental health and community reintegration in veterans.

**Methods**: Fifty‐eight veterans attended psilocybin or ayahuasca retreats. Participants completed eight mental health questionnaires (e.g. PTSD Checklist, PCL‐5; Patient Health Questionnaire, PHQ‐9), and the Military to Civilian Questionnaire (M2C‐Q) up to 4 weeks both pre‐ and post‐retreat. Paired *t*‐tests analyzed changes in outcome responses between time points, and gender and substance‐specific analysis was conducted. Baseline scores were correlated with improvements in PCL‐5 and PHQ‐9 to investigate the relationship between initial symptom severity and percentage improvement following the retreat.

**Results**: Significant improvements were found for all eight outcomes post‐retreat, with the greatest percentage improvements found for depression (PHQ‐9; 29.1%) and PTSD (PCL‐5; 26.1%). Veterans attending psilocybin retreats showed greater improvements in seven out of eight outcomes, whereas ayahuasca retreats demonstrated greater improvements in PCL‐5 scores for PTSD (ayahuasca: 26.4%; Psilocybin 24.8%). Male participants experienced greater improvements across all outcomes apart from the PCL‐5 for PTSD (Male: 24.1%; Female: 32.1%). Higher baseline scores on the PCL‐5 (PTSD) and PHQ‐9 (depression), indicating worse initial mental health, correlated with greater outcome improvements.

**Conclusions**: This is the first study to investigate psychedelic retreats as a holistic therapy for veterans’ mental health alongside community reintegration. Psilocybin and ayahuasca retreats significantly improved veterans’ mental well‐being, quality of life, PTSD, anxiety, depression, sleep, concussion, and post‐deployment reintegration. Participants with more severe symptoms have the potential to benefit most from this intervention, with nuanced insight into improved outcomes based on gender and type of substance. Psychedelic retreats could provide a treatment framework to aid veterans’ recovery by addressing psychological well‐being, communal factors, and reintegration into civilian life.

## Graphical abstract

Military veterans experience higher rates of mental health conditions such as stress disorder (PTSD) and depression, and often face challenges rejoining civilian life after deployment. This study investigated whether attending a communal retreat where they received a psychedelic substance improved the mental health and civilian reintegration of 58 veterans.

Veterans experienced improvements across all outcomes, particularly in depression and PTSD symptoms. Psilocybin mushroom retreats led to broader improvements, while ayahuasca retreats and female participants saw greater PTSD reductions. Veterans with more severe PTSD and depression symptoms before the retreat experienced the greatest improvements in related symptoms. These findings suggest psychedelic retreats may offer a holistic treatment for veterans by addressing psychological well‐being, communal factors and reintegration into civilian life.

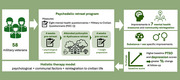

## 1 Introduction

Military veterans represent a population at high risk for mental health issues, particularly post‐traumatic stress disorder (PTSD), often accompanied by comorbidities and reduced overall well‐being (Goodwin et al. 2023; Rhead et al. 2022; Wisco et al. 2022). Traditional treatment options for PTSD, such as psychotherapy—including cognitive behavioral therapy (CBT), cognitive processing therapy (CPT), and eye movement desensitization and reprocessing (EMDR)—consistently yield high dropout, nonresponse, and relapse rates (Edwards‐Steward et al. 2021; Steenkamp et al. 2015). Among veterans with PTSD, dropout rates range from 16% to 35% for CPT, with a rate of 27.1% across trauma‐focused therapies (Edwards‐Steward et al. 2021; Steenkamp et al. 2015). A meta‐analysis of manualized first‐line psychotherapy reported high nonresponse rates, with 50% of military and 35% of civilian participants continuing to meet diagnostic criteria for PTSD following treatment (Straud et al. 2019). In addition, approximately 50% of veterans grappling with PTSD do not access treatment due to barriers such as stigma and limited access to care (Williamson et al. 2022).

Beyond these challenges, pharmacological interventions have poor efficacy and adverse side effects that can be detrimental to the overall health and well‐being of military veterans (Hertzberg et al. 2000; Krystal et al. 2017; Sullivan and Neria 2009). The reliance on pharmacological treatments for veterans with PTSD has faced criticism for prioritizing symptom management over addressing the complex social and psychological dimensions of PTSD amongst veterans (Reisman 2016). Building a supportive community is crucial for veterans to share experiences, promote mutual understanding, and foster a sense of belonging—factors that are integral to the healing process (Balmer et al. 2021; Nichter et al. 2020). The lack of emphasis on community building in psychological interventions can hinder the holistic recovery of veterans. Pharmacology alone may overlook the broader context of veterans’ lived experiences and the unique challenges they face upon returning to civilian life. Given these limitations, integrating community‐building initiatives may offer a more comprehensive approach to PTSD treatment in veterans, acknowledging the interconnected nature of mental health and social support. Psychedelic retreat programs incorporate group‐based sessions as a core element of the treatment model to foster community support and understanding. This holistic approach has the potential to address high rates of dropout and non‐responsiveness among veterans.

The therapeutic potential of psychedelics in treating mental health disorders has gained significant attention in recent years, providing promising, novel avenues for treating conditions like treatment‐resistant depression and PTSD (Kurtz et al. 2022; Mitchell et al. 2021). Psychedelics, including psilocybin, lysergic acid diethylamide (LSD), and *N,N*‐dimethyltryptamine (DMT), are a class of substances that induce altered states of consciousness, often described as mystical experiences eliciting profound insights (Carhart‐Harris and Goodwin 2017; Nichols 2016). These substances, rooted in cultural and spiritual practices (George et al. 2022; Nichols 2016), are believed to address the primary causes of psychological distress by facilitating new perspectives and emotional breakthroughs (Roseman et al. 2019). The pharmacological mechanism of psychedelics is primarily attributed to agonism at serotonin (5‐HT) receptors, particularly the 5‐HT_2A_ receptor subtype (Nichols 2016; van Elk and Yaden 2022), leading to altered patterns of neural activity and enhanced communication between brain regions (De Gregorio et al. 2018; Nichols 2016; Ly et al. 2018). By modulating serotonergic neurotransmission, particularly at the 5‐HT_2A_ receptor, psychedelics such as psilocybin and DMT are proposed to influence mood regulation, emotional processing, and the restructuring of neural pathways associated with trauma (Calder and Hasler 2023; Gasser et al. 2015). The acute psychedelic experience, particularly mystical‐type effects, is thought to moderate positive psychological outcomes (Irrmischer et al. 2025; Yaden and Griffiths 2021). Novel insights, such as an ‘appreciation of life’, have also been identified as mediators of longer‐term improvements in PTSD symptoms (Irrmischer et al. 2025) and important predictors for positive therapeutic outcomes following psychedelic administration (Kugel et al. 2025). This receptor‐mediated mechanism, combined with alterations in brain network activity and enhanced neuroplasticity (e.g. Vargas et al. 2023), offers a compelling framework for understanding the therapeutic potential of psychedelics in fostering profound healing experiences and mitigating the symptoms of various mental health conditions (Ly et al. 2018).

Psilocybin is a naturally occurring psychedelic compound belonging to the mushroom genus Psilocybe (Passie et al. 2002). Its chemical structure is similar to serotonin, a neurotransmitter that plays a crucial role in mood regulation (Lowe et al. 2021). Preliminary research supports the effectiveness of psilocybin‐assisted therapy in treating various mental health conditions, including treatment‐resistant depression, anxiety, and substance dependence disorders (Bogenschutz et al. 2015; Carhart‐Harris and Goodwin 2017). Comparably, ayahuasca is a psychedelic decoction from the *Banisteriopsis caapi* vine combined with the leaves of the *Psychotria viridis* shrub, in which DMT is the primary psychoactive compound (McKenna et al. 1984). Indigenous cultures have used ayahuasca in traditional Amazonian healing practices for centuries (Labate and Cavnar 2013). Preliminary studies with ayahuasca in both clinical and naturalistic settings have shown promising results in treating PTSD and depression (Osório et al. 2015; Palhano‐Fontes et al. 2019; Weiss, Dinh‐Williams, et al. 2023).

Psychedelic medicines have been explored to address the needs of veterans by leveraging their transformative qualities and potential effectiveness in managing psychological disorders. Initial studies have suggested that a psychedelic experience in a retreat setting can lead to significant reductions in symptoms of PTSD, depression, and anxiety among veterans (Davis et al. 2020; Davis, Xin, et al. 2023; Weiss, Dinh‐Williams, et al. 2023).

The communal nature of retreat settings has been suggested as a crucial element in enhancing the therapeutic impact of psychedelics (Kettner et al. 2021), fostering opportunities for shared experiences, mutual support, and a sense of camaraderie– elements that could be particularly beneficial for veterans who report isolation post‐service (Nichter et al. 2020). Psychedelic retreat programs, where veterans enroll as cohorts, emerge as a potential avenue not only to alleviate PTSD symptoms but also to contribute to the broader goal of fostering the well‐being of veterans through shared experiences and group support. Furthermore, ceremonial retreats offer a unique therapeutic framework for addressing PTSD by combining the pharmacological effects of psychedelics with ritualistic and communal elements that enhance emotional processing and integration (Weiss, Dinh‐Williams, et al. 2023). These ceremonial therapeutic settings provide a holistic approach that may offer veterans a comprehensive framework for healing that aligns with their experiences and needs.

In addition, administering psychedelic medicine in retreat programs offers immersive and holistic therapeutic experiences. These programs typically entail veterans consuming the psychedelic substance over a residential period at a retreat center over around 5–7 days (Orozco and Harris 2023). Beyond the pharmacological aspect, the retreat experience incorporates a range of supportive activities and discussions designed to enhance the therapeutic impact (Kettner et al. 2021). Experienced guides play a crucial role during the retreat, providing support and guidance to help veterans navigate their psychedelic experience (Davis, Xin, et al. 2023). These guides are trained facilitators who support participants before, during, and following the psychedelic session, ensuring safety and providing emotional support as well as encouraging meaningful reflection (Kinahan and Wilson 2025). Preparation sessions held prior to the retreat contribute significantly to the overall effectiveness of psychedelic therapy (Modlin et al. 2023). These sessions, run by guides, involve thorough discussions about the upcoming experience, the potential challenges that may arise, and the intentions the veterans have for participating in the retreat. Establishing a sense of trust and safety is paramount during this preparatory phase, ensuring that participants are mentally and emotionally prepared, and guides play a key role in building a strong therapeutic relationship (Gorman et al. 2021; Kinahan and Wilson 2025). Integration sessions held following the retreat are equally crucial (Gorman et al. 2021). These sessions provide a structured environment for veterans to reflect on and make sense of any potential insights from their psychedelic experiences. Integrative therapy aims to help veterans apply these newfound perspectives and understandings to their daily lives, promoting long‐term positive changes (Watts and Luoma 2020). In facilitated group sessions, veterans share this experience with others in their retreat cohort, cultivating a sense of community and understanding among individuals who have faced similar challenges. The combination of immersive retreat experiences, peer support, and thoughtful preparation and integration phases likely contribute to the enduring therapeutic effects of psychedelic therapy for PTSD in veterans. However, research in this field is ongoing (Biscoe et al. 2023; Davis, Levin, et al. 2023) and more comprehensive research is needed to better understand the full potential of psychedelic retreats when delivered to groups of veterans experiencing psychological distress. This observational study aims to advance our understanding of how psychedelic retreat programs improve symptoms associated with psychological distress and address mental health challenges experienced by veterans.

It is important to acknowledge the risks and side effects of psychedelic administration in veteran populations. Although substance use disorder is prevalent among veterans (e.g., Teeters et al. 2017), evidence indicates that psychedelics are non‐addictive substances (Nichols 2016) and even show promise as treatments for addictive disorders (Zafar et al. 2023). This suggests that there is a low risk of dependence on psychedelics following the retreat. Moreover, adverse side effects are generally short‐lived, subsiding after the subjective experience (Perez et al. 2023). Nonetheless, adverse psychological reactions, including panic responses, can occur and must be managed effectively with psychological support (Perez et al. 2023).

This study introduces a novel dimension to the existing body of research by utilizing the Military to Civilian Questionnaire (M2C‐Q), which is designed to measure the challenges and problems veterans face when transitioning from military service to civilian life, including social integration, employment, and mental health (Sayer et al. 2011). The transition from military service to civilian life is a vital phase for fostering overall mental and physical well‐being, as it involves adjustments in identity, daily routines, social networks, and support systems (Adler et al. 2011; N. Rattray et al. 2023). Difficulties in this transition can lead to isolation, unemployment, and exacerbate mental health difficulties, particularly PTSD and depression (N. A. Rattray et al. 2019). Successfully navigating this phase is essential for veterans to lay the foundation for overall mental and physical health, purpose, and community connection (N. Rattray et al. 2023; Thompson et al. 2022). While difficulties with reintegration are not common, they are often reported by the subset of veterans with disorders such as PTSD and traumatic head injuries (Sayer et al. 2015). In addition, this study further advances the field by interrogating the relationship between baseline scores and overall change in scores across the battery of questionnaires employed. This comprehensive approach facilitates analysis of the therapeutic potential of psychedelics for treating psychological distress among veterans extending beyond symptom relief to encompass broader social and psychological well‐being. Thus, this study pioneers an integrated retreat framework with the potential to reshape psychedelic retreat paradigms to effectively assist in military veteran rehabilitation. We hypothesize participation in psychedelic retreats will have a significant, positive effect on all measures of psychological distress and civilian life integration amongst veterans, and that participants with the least optimal scores prior to the retreat will exhibit the largest improvements.

## 2 Methods

### 2.1 Participants

Participants were invited to take part in this research after confirming attendance at a psychedelic retreat program hosted by Heroic Hearts Project, a charity providing psychedelic retreat programs to veterans suffering from psychological distress or experiencing negative health outcomes. Potential research participants were informed of the research via email where they were able to self‐enroll in the study through an online link. All participants provided informed consent. Ethics approval was granted by the Joint Research Compliance Office and the Imperial College Research Ethics Committee (ICREC reference 18IC4346).

Retreat attendees completed comprehensive medical and psychological screening for eligibility in the retreat. This ensured they were in good general health and medication‐naïve to all antidepressants, antipsychotic medications, monoamine oxidase inhibitors, and dietary supplements that include 5‐hydroxytryptophan and St John's wort. Participants had no reported history of psychotic disorders or borderline personality disorder, no first‐degree relative diagnosed with a psychotic disorder, and did not present in a psychological state deemed unsuitable by Heroic Hearts Project's screening staff.

### 2.2 Procedure

**FIGURE 1:**
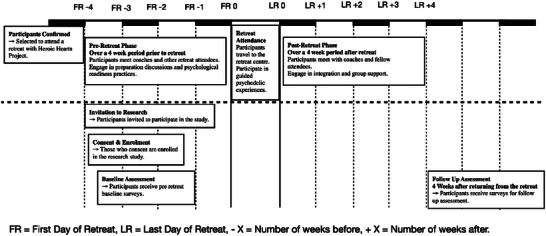
Schedule of psychedelic retreat and research stages.

Figure 1 displays the retreat program and research schedule. Participants were sent pre‐retreat surveys to collect demographic information and mental health questionnaire scores at baseline — up to 4 weeks before leaving for the retreat. Participants who completed the retreat program were sent follow‐up questionnaires to complete 4 weeks after they returned from the retreat and had completed all group and individual integration sessions. All surveys were collected online through Alchemer and participants received individualized links to the survey, at the time required for completion.

### 2.3 Measures

The following questionnaires were used to assess a variety of psychological distress measures. The Patient Health Questionnaire (PHQ‐9) is a diagnostic tool used to assess the severity of depression with a diagnostic threshold of 10 or higher indicating clinically significant depression (Kroenke et al. 2001). The Quality of Life after Brain Injury (QOLIBRI; Von Steinbüchel et al. 2010) measured the quality of life specifically in individuals who have suffered a traumatic brain injury (TBI). The Rivermead Post‐Concussion Symptom Questionnaire (RPQ; King et al. 1995) evaluated the severity of post‐concussion symptoms. The Posttraumatic Stress Disorder Checklist (PCL‐5; Blevins et al. 2015) screened for PTSD and measured its severity, with a score of 31–33 or higher suggesting a PTSD diagnosis (Bovin et al. 2016). The PROMIS Sleep Disturbance short form (PROMIS; Yu et al. 2011) assessed sleep quality and disturbances. The Short Warwick‐Edinburgh Mental Wellbeing Scale (SWEMWBS; Ng Fat et al. 2017) measured mental well‐being and positive mental health. The State‐Trait Anxiety Inventory (STAI‐T; Spielberger et al. 1970) evaluated trait anxiety, with scores of 40 or above indicating clinically significant symptoms of anxiety (Emons et al. 2019). Finally, the M2C‐Q is designed to assess social relations, productivity in education, work and domestic life, community engagement and perceived meaning in life, self‐care, and leisure in the transition experiences of military personnel moving to civilian life (Sayer et al. 2011). The M2C‐Q was specifically constructed for use with US military veterans with post‐deployment reintegration difficulties (Sayer et al. 2011).

Table 1 shows the attributes of each measure. This includes the score range, scale, diagnostic threshold, and directionality which are used to derive percent improvement.

**TABLE 1:** Summary of measures used in the study.

Measure	Formal name	Topic	Score range	Scale	Diagnostic threshold	Directionality
PCL‐5	PTSD checklist	PTSD	0–80	80	31–33	High denotes adverse status
PHQ‐9	Patient Health Questionnaire	Depression	0–27	27	10	High denotes adverse status
STAI‐T	State‐Trait Anxiety Inventory	Anxiety	20–80	60	40	High denotes adverse status
RPQ	Rivermead Post‐Concussion Symptoms	Concussion	0–53	53		High denotes adverse status
PROMIS	Patient‐Reported Outcomes Measurement Information System	Sleep	20–80	60		High denotes adverse status
M2C‐Q	Military to Civilian Questionnaire	Civilian Reintegration	0–4	5		High denotes adverse status
QOLIBRI	Quality of Life After Brain Injury	Quality of Life	1–100	100		High denotes favorable status
SWEMWBS	Short Warwick‐Edinburgh Mental Wellbeing Scales	Mental Wellbeing	14–70	56		High denotes favorable status

### 2.4 Retreat

Participants were invited to complete three individual and three group coaching sessions both prior to traveling to retreat centers and after the retreat (Figure 1). The coaching sessions were conducted remotely and provided by the Heroic Hearts Project. The group coaching sessions were composed of participants attending the same retreat. The retreats were all held internationally, requiring the participants to travel out of the country to attend. These were held in Jamaica for those who took psilocybin and in Peru for those who took ayahuasca. The retreat, and therefore substance, participants were assigned to, was dependent on retreat availability at the time of enrolment. Participants attending ayahuasca retreats took the substance orally as a liquid with a dosage determined by the tribe and titrated by the individual during ceremonies to elicit a full psychedelic experience. For psilocybin, the substance was taken as a tea brewed from dried psilocybin mushrooms with individualized doses determined by the retreat staff between 1.5 and 3.5 g for Session 1 and between 3 and 5 g for Session 2. One gram boosters of psilocybin were offered one hour from the initial dose. The ayahuasca group participated in three consecutive ceremonies of 6–10 h, whereas those administered psilocybin completed two ceremonies 48 h apart. Participants were required to stay at the retreat site for the duration of the retreat.

### 2.5 Analysis

All data were analyzed using SPSS 28.0 (14) (IBM 2021). In the case where a value was missing from the dataset, the participant was excluded from that analysis. One participant had missing data for the STAI‐T and M2C‐Q questionnaires and was excluded from these analyses. Frequency counts and descriptive analyses of demographic characteristics and mental health history were conducted. Paired‐samples two‐tailed *t*‐tests were used to compare mean changes in self‐reported scores between baseline and follow‐up across multiple dimensions, including depression, anxiety, PTSD, quality of life after brain injury, post‐concussion symptoms, physical, mental, and social well‐being, and post‐deployment reintegration. An alpha of 0.05 was used to determine statistical significance. The effect sizes were calculated using Cohen's *d* and were considered medium when *d* ≥ 0.5, large when *d* ≥ 0.8, and very large when *d* ≥ 1.2 (Cohen 1992). The Holm–Bonferroni procedure was applied to correct for multiple comparisons (Holm 1979). Post‐hoc power calculations were conducted for the paired *t*‐tests to determine whether the sample size was sufficient to detect observed effect sizes with adequate statistical power.

The questionnaire scores were on differing scales. In other words, 5 point improvement on the PCL‐5 may not be directly comparable to 5 points improvement on the PHQ‐9. To permit results comparison, we normalized all scores to a consistent measure of percent improvement. We derived QOLIBRI and SWEMWBS conversely since their directionality is the opposite of the other measures. Finally, we derived percent improvement as follows:

$$\begin{array}{ccc} & & \left(\mathrm{Baseline}-\mathrm{endpoint})/\mathrm{scale}\;(\mathrm{if}\;a\;\mathrm{higher}\;\mathrm{score}\;\mathrm{is}\;\mathrm{worse}\right)\\ & & \;\;\;\;\;\;\mathrm{or},\\ & & (\mathrm{Endpoint}-\mathrm{baseline})/\mathrm{scale}\;(\mathrm{if}\;a\;\mathrm{higher}\;\mathrm{score}\;\mathrm{is}\;\mathrm{better}). \end{array}$$

To investigate differences in percentage change in scores between retreat types (ayahuasca and psilocybin), gender, and those with a PTSD diagnosis, we descriptively analyzed the total improvement in all measures separately for each group. Analyses explored potential trends in retreat responsiveness rather than testing for statistically significant differences between subgroups. We then visualized baseline scores against improvement in scores for the two measures with the greatest average percent improvement (separated by substance and gender) to assess if there was a relationship.

### 2.6 Post‐Hoc Analysis

Exploratory analysis was conducted to examine the relationship between baseline scores and percent improvement for the two measures with the greatest percent improvement using simple linear regression analyses.

## 3 Results

### 3.1 Participants Demographics

Data was collected from 58 participants, 44 males (75.9%) and 14 females (24.1%), aged between 26 and 69 (Mean = 40.86, SD = 7.99, 95% CI [38.76, 42.97]). Of the 58 participants, 47 reported a diagnosis of PTSD (81%). Forty‐five participants attended an ayahuasca retreat (77.6%) and 13 attended a psilocybin retreat (22.4%). In addition, 16 participants had never taken a psychedelic prior to the retreat program (32.5%). Table 2 describes the demographic statistics.

**TABLE 2:** Participant demographics (*N* = 58).

Participant demographics	*N* = 58	
**Age (years)**		95% CI
Mean (SD)	40.86 (7.99)	[38.76, 42.97]
**Gender**		**%**
Male	44	75.9
Female	14	24.1
**Nationality**		
United States	51	87.9
United Kingdom	5	8.6
Canada	1	1.7
Philippines	1	1.7
**Highest Education**		
Graduated high school	5	8.6
Trade/technical school	4	6.9
Some college, no degree	12	20.7
Bachelor's degree	23	39.7
Master's degree	13	22.4
Doctorate or professional degree	1	1.7
**Employment status**		
Employed full time (40 or more hours/week)	19	32.8
Employed part time (up to 39 h/week)	3	5.2
Self‐employed	12	20.7
Student	5	8.6
Homemaker	2	3.4
Unemployed	6	10.3
Unable to work	3	5.2
Retired	8	13.8
**Marital status**		
Married	28	48.3
In a relationship	3	5.2
Single (never married)	13	22.4
Divorced	12	20.7
Separated	1	1.7
Widowed	1	1.7
**Reported mental health disorder diagnosis**		
Major depressive disorder	28	48.3
Anxiety disorder (e.g., OCD)	21	53.4
Post‐traumatic stress disorder (PTSD)	47	81
Substance use disorder	10	17.2
Alcohol dependence	11	19
Eating disorder	2	3.4
ADHD	19	32.8
Autism spectrum disorder	1	1.7
Phobia	1	1.7
Chronic pain	39	67.2
**Number of times taken a classic psychedelic (LSD, psilocybin, DMT)**		
Never	16	27.6
Only once	5	8.6
2–5 times	16	27.6
6–10 times	6	10.3
11–20 times	4	6.9
21–50 times	8	13.8
51–100 times	2	3.4
More than 100 times	1	1.7
**Baseline attitude pre‐retreat: “I am an active advocate of the therapeutic use of psychedelics/plant medicines”**		
Strongly disagree	0	0
Disagree	1	1.7
Neither agree nor disagree	5	8.6
Agree	13	22.4
Strongly agree	39	67.2
**Ceremony Type**		
Ayahuasca	45	77.6
Psilocybin	13	22.4

Abbreviations: ADHD, attention deficit hyperactivity disorder; DMT, *N, N*‐dimethyltryptamine; OCD, obsessive compulsive disorder; SD, standard deviation.

### 3.2 Veterans Showed Significant Improvements Across All Well‐Being Measures

Analyses examined whether participation in psychedelic retreats was associated with changes in the different measures utilized (Table 3). Individual questionnaire baseline and endpoint retreat scores are presented in Supporting Information (Figures S1–S8), alongside the distribution of percentage improved across participants (Figure S9). Paired‐sample *t*‐tests revealed participation in psychedelic retreat programs was associated with significant improvements on all measures, with medium to very large effect sizes. The study revealed significant, large improvements in scores for depression measured by the PHQ‐9 (*p* < 0.001, SD = 6.70, 95% CI [6.09, 9.61], Figure S1), PTSD measured by the PCL‐5 (*p* < 0.001, SD = 18.68, 95% CI [15.90, 25.72], Figure S4), post‐concussion symptoms measured by the RPQ (*p* < 0.001, SD = 14.36, 95% CI [8.41, 15.97], Figure S3), mental wellbeing measured by the SWEMWBS (*p* < 0.001, SD = 4.96, 95% CI [3.50, 6.12], Figure S6), and post‐deployment reintegration measured by M2C‐Q (*p* < 0.001, SD = 0.84, 95% CI [0.65, 1.10], Figure S8) between baseline and post‐retreat responses. In addition, the analysis found very large improvements in anxiety measured by the STAI‐T (*p* < 0.001, SD = 10.19, 95% CI [11.03, 16.44], Figure S7) and quality of life after a TBI measured using the QOLIBRI (*p* < 0.001, SD = 16.06, 95% CI [18.41, 26.85], Figure S2). Lastly, there was a significant and medium improvement in sleep as measured by PROMIS (*p* < 0.001, SD = 11.91, 95% CI [5.29, 11.56], Figure S5).

**TABLE 3:** Primary outcome measures (*N* = 58).

	Baseline *M* (SD)	Endpoint *M* (SD)	Change *M* (SD) [95% CI]			
Measure				*p* value	*t* (df)	Cohen's *d*
**PHQ‐9**	13.43 (5.77)	5.59 (4.59)	7.85 (6.70) [6.09, 9.61]	<0.001	8.93 (57)	1.172
**QOLIBRI**	43.79 (13.62)	66.41 (17.03)	22.63 (16.06) [18.41, 26.85]	<0.001	10.73 (57)	1.409
**RPQ**	27.52 (14.24)	15.33 (12.43)	12.19 (14.36) [8.41, 15.97]	<0.001	6.46 (57)	0.849
**PCL‐5**	39.95 (16.51)	19.14 (16.25)	20.81 (18.68) [15.90, 25.72]	<0.001	8.49 (57)	1.114
**PROMIS**	59.92 (8.80)	51.493 (10.80)	8.43 (11.91) [5.29, 11.56]	<0.001	5.39 (57)	0.707
**SWEMWBS**	18.77 (3.10)	23.57 (4.96)	4.81 (4.96) [3.50, 6.12]	<0.001	7.38 (57)	0.969
**STAI‐T**	54.04 (9.61)	40.30 (11.74)	13.74 (10.19) [11.03, 16.44]	<0.001	10.18 (56)	1.348
**M2C‐Q**	1.89 (0.76)	1.02 (0.80)	0.88 (0.84) [0.65, 1.10]	<0.001	7.82 (56)	1.036

*Note*: Cohen's *d* (medium *d* ≥ 0.5, large *d* ≥ 0.8 and very large *d* ≥ 1.2).

Abbreviations: M2C‐Q, Military to Civilian Questionnaire; PHQ‐9, Patient Health Questionnaire; PLC‐5, PTSD checklist; PROMIS, Patient‐Reported Outcomes Measurement Information System; QOLIBRI, Quality of Life After Brain Injury; RPQ, Rivermead Post‐Concussion Symptoms; STAI‐T, State‐Trait Anxiety Inventory; SWEMWBS, Short Warwick‐Edinburgh Mental Wellbeing Scales.

### 3.3 Percentage Improvement Varied by Substance and Gender

**FIGURE 2:**
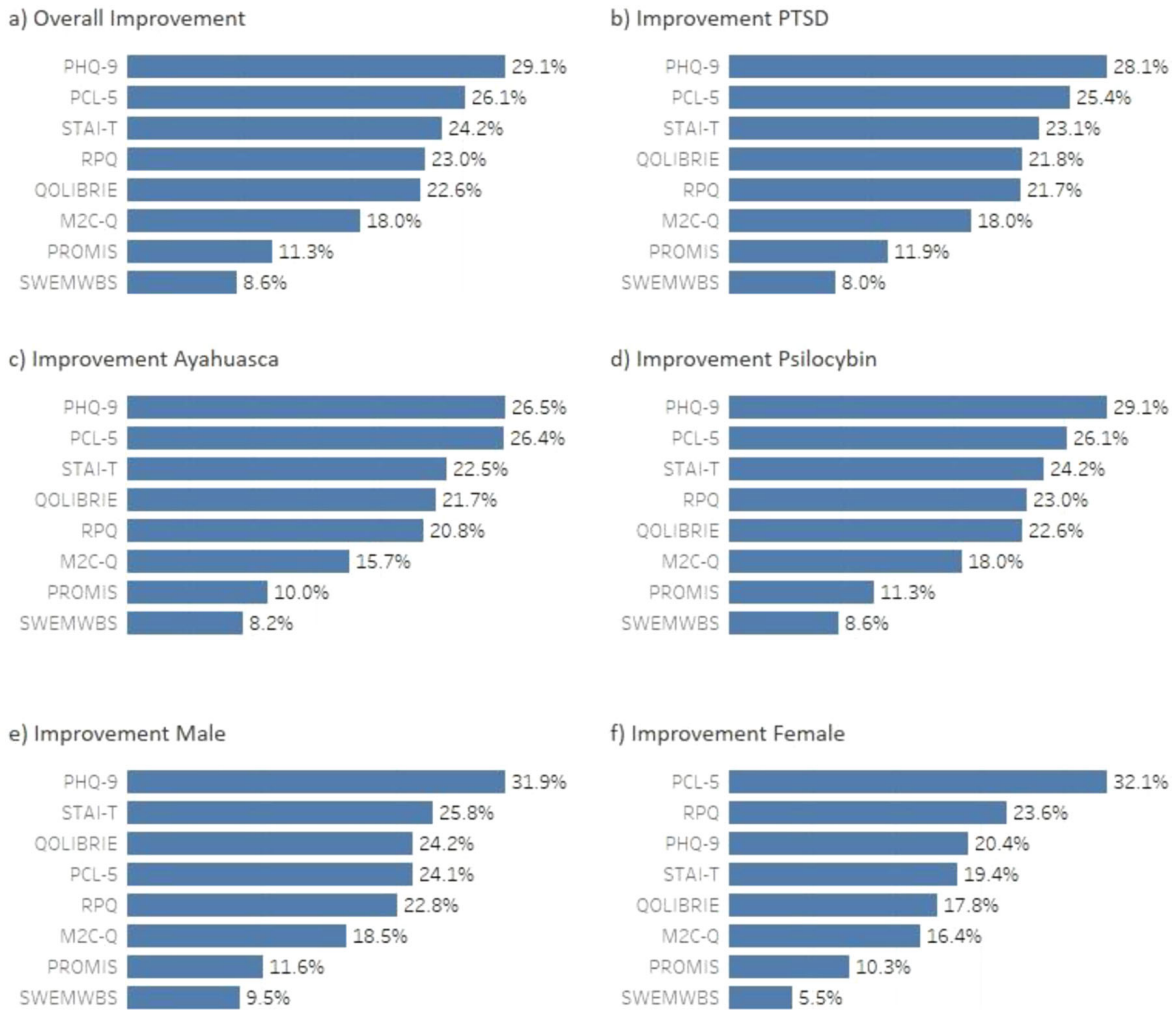
Average percent improvement for (a) overall improvement, (b) in PTSD scores measured with the PCL‐5, (c) of participants on Ayahuasca retreats, (d) of participants on psilocybin retreats, (e) males only, and (f) females only.

Analysis of percentage improvement for each score (Figure 2a‐f) demonstrated the overall average percent improvement in responses for all participants (a), those with a PTSD diagnosis (b), those who attended ayahuasca retreats (c), those who attended psilocybin retreats (d), those who were male (e), and those who were female (f).

Results show participants with a reported PTSD diagnosis did not experience greater improvements than the general cohort across the measures used. Participants who attended psilocybin retreats exhibited greater reductions in depression (PHQ‐9: 38% improvement compared to 26.5% for ayahuasca), anxiety (STAI‐T: 30.4% improvement compared to 22.5% for ayahuasca), post‐concussion symptoms (RPQ: 30.5% improvement compared to 20.8% for ayahuasca), and post‐deployment reintegration (M2C‐Q: 25.8% compared to 15.7% for ayahuasca; Figure 2c,d). However, participants who attended the ayahuasca retreats showed a slightly higher improvement in PTSD symptoms (26.4%) compared to those in psilocybin retreats (24.8%).

Gender‐specific analyses (Figure 2e,f) revealed male participants generally showed greater improvements in measures of depression (26.5% improvement compared to 20.4% for females), anxiety (25.8% improvement compared to 19.4% for females), quality of life after a brain injury (24.2% improvement compared to 17.8% for females), and civilian reintegration (18.5% improvement compared to 16.4% for females). However, female participants exhibited a greater improvement in PTSD symptoms (32.1%) compared to male participants (24.1%).

### 3.4 Higher PHQ‐9 and PCL‐5 Scores Were Associated With Greater Percentage Improvements

**FIGURE 3:**
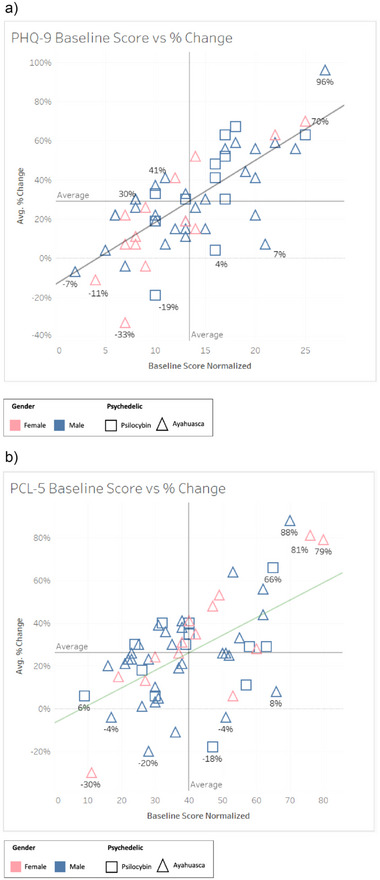
Relationship Between Baseline Scores and Percent Improvement for PHQ‐9 and PCL‐5. Baseline score versus percent improvement for (a) PHQ‐9 and (b) PCL‐5 scores, the two measures with greatest average percent improvement. Participant distribution by gender (color) and substance (shape). Participants with higher baseline scores, indicating worse mental health, exhibited greater percentage improvements. PLC‐5: PTSD Checklist; PHQ‐9: Patient Health Questionnaire.

Baseline scores were plotted against percentage change in scores for the two measures with the greatest average percent improvement, PCL‐5 and PHQ‐9 measures of PTSD and depression, separated by substance and gender (Figure 3). Figure 3a,b shows participants with higher baseline scores (indicating worse initial mental health) tended to experience greater improvements at the endpoint. Conversely, participants with lower baseline scores (indicating better initial mental health) saw more modest improvements. This trend was consistent across genders and types of psychedelic substances. Post‐hoc regression analysis revealed a significant relationship between baseline scores and percentage improvement for the PHQ‐8 (*R* ^2^ = 0.544, *p* < 0.001) and PCL‐5 (*R* ^2^ = 0.336, *p* < 0.001).

### 3.5 Post‐Hoc Power Analysis

Post‐hoc power analyses conducted using G*power (v3.1) for the paired *t*‐tests revealed high statistical power for all outcome measures (>0.99, α = 0.05). The high power was primarily driven by large observed effect sizes and adequate sample size (Table 3).

## 4 Discussion

This is the first study investigating the effects of psychedelic retreat programs for military veterans alongside community integration into civilian life, finding significant improvements across all outcome measures related to psychological and general well‐being four weeks after retreat attendance. Participants who attended a Heroic Hearts Project psychedelic retreat program experienced significant improvements in general health, well‐being, perceived quality of life, anxiety, depression, sleep, and post‐deployment reintegration. In addition, attending a psychedelic retreat had large, significant effects on self‐reported measures of PTSD. To further assess the impact of the retreat intervention on veterans specifically, we used the Rivermead Post‐Concussion Symptom Questionnaire (RPQ), to measure physical and psychological symptoms associated with head trauma, as head traumas and exposure to blast force are commonplace in the military (Phipps et al. 2020). A large significant improvement in symptoms associated with head trauma was found following retreat attendance. Overall, findings underscore the positive impact of the psychedelic retreat program on various aspects of veterans' mental health and general well‐being.

Results align with previous research that has demonstrated the therapeutic potential of psychedelic medicines for treating mental health conditions (Carhart‐Harris and Goodwin. 2017; Reiff et al. 2020) and improving general psychological well‐being (Mans et al. 2021). However, beneficial effects were not investigated beyond the 4‐week follow‐up, limiting insight into whether changes in outcome measures were enduring. Findings support research conducted with veterans in psychedelic retreat programs that have shown significant reductions in PTSD, depression, and anxiety (Davis, Levin, et al. 2023; Davis, Xin, et al. 2023; Weiss, Dinh‐Williams, et al. 2023). Positive effects observed following the administration of psychedelics in communal naturalistic or retreat settings (Ruffell et al. 2021) could highlight the importance of the group experience in therapeutic outcomes (Kettner et al. 2021; Kiraga et al. 2022). This is particularly relevant for veterans, as social support is a crucial aspect of the healing process within veteran communities as they return to civilian life (Nichter et al. 2020). The communal aspect of psychedelic retreats may therefore enhance the therapeutic benefits in this group by creating the shared experience and support essential for veterans' reintegration, though further research on the unique contribution of social and group factors is required. This research is particularly warranted given the effectiveness of psychedelics in improving veteran mental health in non‐retreat settings (Ellis et al. 2025).

Participants with a reported PTSD diagnosis did not demonstrate greater improvements than the general cohort across outcomes. While significant improvements were observed in PTSD symptoms for the PTSD sub‐group, these were comparable to improvements seen in participants without a PTSD diagnosis. This suggests the observed benefits of the psychedelic retreat are widespread and not limited to participants with specific clinical diagnoses, making the intervention broadly applicable to veterans facing various psychological challenges. Mechanistically, the psychedelic retreat could facilitate holistic psychological shifts underlying general therapeutic benefits, rather than specifically targeting PTSD pathology. This aligns with research describing the effects of psychedelic retreats as transformative (Neitzke‐Spruill et al. 2024) and resulting in improved mindfulness and psychosocial functioning (Smigielski et al. 2019), indicating a broader therapeutic mechanism explained through enhancing general well‐being.

This study was the first, to our knowledge, to utilize the M2C‐Q to evaluate the effects of attending a psychedelic retreat program on veterans' reintegration into civilian life. The M2C‐Q specifically measures challenges related to social integration, employment, and overall functioning during the transition from military to civilian contexts (Sayer et al. 2011). By utilizing this measure, our research provides novel insights into how psychedelic retreats can positively impact broader aspects of veterans' reintegration. The study revealed significant improvements in veterans’ reintegration into civilian life following a psychedelic retreat, with an overall 18% improvement in M2C‐Q scores. These improvements highlight the potential of psychedelic retreat programs to support the complex process of veterans’ reintegration into civilian life. This could provide a novel therapeutic approach as veteran reintegration is essential to establish their overall well‐being, purpose, and community (Balmer et al. 2021; N. Rattray et al. 2023; Thompson et al. 2022). However, further studies should delineate the relative contribution of pharmacological factors, from psychedelic medicine, and supportive group settings in veteran reintegration. Interpersonal and community challenges are common barriers to reintegration in this group (Elnitsky et al. 2017), which may have been addressed by the communal component of the retreat including shared experiences and group preparation sessions, independent of psychedelics’ pharmacological action. Nonetheless, psychedelic retreats can offer a treatment paradigm that enhances veterans’ overall well‐being and transition into civilian life by addressing both psychological distress and the broader social challenges associated with reintegration in a single framework.

Comparing ayahuasca and psilocybin retreats, participants who were administered psilocybin exhibited greater reductions in depression, anxiety, post‐concussion symptoms, and post‐deployment reintegration. Contrastingly, participants in the ayahuasca retreats showed a slightly greater improvement in PTSD symptoms. This suggests that, while psilocybin may be more effective for treating a wide range of conditions and challenges faced by veterans, ayahuasca could offer specific benefits for treating PTSD. Previously, in an ayahuasca retreat setting, veterans and participants with PTSD re‐experienced traumatic events at a higher prevalence than non‐veterans and participants without a PTSD diagnosis (Weiss, Wingert, et al. 2023). Authors proposed that the re‐experiencing of traumatic events was associated with psychological healing mechanisms, such as seeing new positive meaning in adverse experiences (Weiss, Wingert, et al. 2023), supporting the beneficial effect of ayahuasca retreat attendance on PTSD symptoms observed in the present study. However, trials investigating the safety and efficacy of both psilocybin and ayahuasca for treating PTSD are lacking. Results highlight the importance of accounting for veterans’ individual mental health needs to inform the optimal psychedelic treatment choice and develop tailored clinical approaches.

Gender‐specific analyses revealed male participants generally showed greater improvements in depression, anxiety, quality of life after a brain injury, and civilian reintegration compared to female participants. However, the low number of female participants (*n* = 14; 24.1%) could suggest results do not represent a generalizable effect size in this population, reflecting a wider need for female representation in psychedelic studies. Female participants did show a greater improvement in PTSD symptoms compared to male participants. Gender differences in responsiveness to psychedelics across psychiatric outcomes could be due to sex‐specific action and pharmacokinetics (Shadani et al. 2024). This finding highlights the importance of considering gender differences in response to psychedelic therapy and for further research investigating how biological sex influences psychedelic responsiveness between outcome measures.

Percentage improvement of PHQ‐9 and PCL‐5 scores (the two outcomes with the greatest average percent improvement) revealed participants with more severe symptoms of depression and PTSD at baseline exhibited more substantial percentage improvements following the retreat. This corroborates findings that higher depression and anxiety levels before psychedelic intervention were associated with greater improvements in several psychological well‐being measures, including symptoms of trauma, depression, and life satisfaction, in veterans after one month (Xin et al. 2023). Moreover, older participants with poorer mental health have been found to experience greater benefits from psychedelic intervention (Kettner et al. 2024). Participants with less severe symptoms at baseline still generally benefited from the retreat but showed less pronounced percentage improvements in PHQ‐9 and PLC‐5 scores. This pattern suggests that the severity of initial symptoms could determine the degree of benefit obtained from the psychedelic retreat and that this intervention is particularly suited for veterans suffering from severe PTSD and depression. Given that veterans with severe PTSD are more likely to avoid or drop out of current interventions requiring continual engagement (Smith et al. 2019; Wells et al. 2023), the therapeutic value of a one‐off retreat intervention could offer significant benefits for veterans with severe symptoms.

### 4.1 Limitations

A key methodological limitation of this study is the absence of a placebo or control condition. This arises from the naturalistic design in which veterans self‐selected for a real‐world retreat, making randomization or control conditions unfeasible. The absence of placebo control significantly limits our ability to separate the potentially confounding therapeutic effect of the natural, communal setting of the retreat. Although social support is essential for the healing and reintegration of veterans (Nichter et al. 2020), the therapeutic contribution of a communal setting is not isolated from the psychedelic experience itself or pharmacological action in this intervention. Given the illegal status of psychedelics in many parts of the world, it is essential to separate the efficacy of pharmacological and communal mechanisms on the specific well‐being outcomes measured to support wide‐spread practical application and accessibility (e.g., providing alternative psychedelic or communal approaches for reintegration vs. depression). Future studies could incorporate a matched comparison group attending a wellness retreat without psychedelics, or collect subjective reports of the perceived impact of the communal setting, to delineate pharmacological and contextual factors. Moreover, expectancy effects, defined as a participant believing they will have a treatment response, are a challenge in psychedelic research and may significantly contribute to therapeutic outcomes (Muthukumaraswamy et al. 2021; Szigeti and Heifets 2024). This is particularly given the lack of a control group and selection bias from self‐enrolment, as demonstrated by participant's high belief in the effectiveness of psychedelic therapies pre‐retreat (Table 2), further highlighting the importance of a placebo‐controlled design.

A related limitation is the inability of the principal analysis, *t*‐tests, to control for other variables likely to influence treatment outcomes, for example, prior psychedelic experience, major life events between assessment time points, and non‐psychiatric medications. We suggest further studies including multiple assessment periods or control groups utilize analyses such as ANCOVA or regression which can account for the influence of covariates.

In addition, adverse events during the psychedelic experience were not recorded to evaluate substance tolerability and safety. Doses taken by each participant were also not recorded, limiting the study's ability to identify a dose‐response relationship with therapeutic or psychological outcomes or any adverse effects. Without systematic monitoring of adverse events or doses, assessing the magnitude and consistency of observed positive effects and the intervention's safety profile is limited. The psychedelic dose has consistently correlated with the intensity of acute subjective experiences (Hirschfeld and Schmidt 2021) and mystical‐type experiences, which are proposed mechanisms underpinning positive outcomes (Yaden and Griffiths 2021). Alternatively, individualized dosing tailored by retreat facilitators may be beneficial to adapt psychedelic dosage based on participant physiology and prior experience. However, the lack of standardized dosing limits the future replication of this study. Future research should incorporate doses to explore relationships between dose and outcome improvements in this specific sub‐group and expand applicability in determining optimal dosing strategies for future applications.

The study collected self‐report data provided by a small sample lacking in diversity, therefore limiting the generalizability of findings. Due to the observational nature of this research, follow‐up was only completed with a subset of participants, which introduces the possibility that positive outcomes reported are associated with a higher willingness to complete the research. Therefore, symptom improvements could be amplified by expectancy effects common in psychedelic research (Butler et al. 2022).

Furthermore, study endpoints were taken after the integration period, with a minimum of four weeks after leaving the retreat. While this time period provides insight into short‐term effects, it does not capture the potential long‐term sustainability of the improvements, as benefits observed across outcomes may subside after 4 weeks. Some measures, such as the M2C‐Q, ask participants to reflect on their experiences over the four weeks prior to the assessment. It is therefore unclear whether the positive effects observed are maintained in the months following the retreat. Long‐term follow‐up with participants would be essential to determine the durability of the observed benefits and to understand the potential for any relapse or continued improvement over time. For example, response and remission rates for veterans with depression reduced from 3 to 12 weeks after psilocybin therapy (Ellis et al. 2025), indicating improvements in mental health may not be robust over time. Overall, limitations highlight the need for more rigorous and comprehensive research methodologies to validate and build upon the findings of this study.

### 4.2 Future Directions

Preliminary results presented in this study highlight the need for further research into psychedelic retreats for veteran mental health to validate the improvements in psychological and general well‐being observed. It is crucial to open the path for novel treatments for those experiencing psychological distress following deployment. Particularly effective therapeutic approaches for PTSD, prevalent in this population, exhibit high drop‐out and nonresponse rates (Edwards‐Steward et al. 2021; Steenkamp et al. 2015). To enhance the reliability and generalizability of the findings, future studies should employ random sampling methods for post‐retreat analysis. This approach would allow for a more comprehensive assessment of the retreat's impact on mental health outcomes, including investigating correlations between participants' willingness to complete the research, indicative of higher expectancy, and reported experiences. Moreover, collecting dose data, exploring a gender‐balanced population, and comparing retreat conditions to a non‐communal control group could provide further insight into mechanisms underpinning observed therapeutic outcomes.

The short‐term assessment of beneficial outcomes highlights the need for future studies with more frequent and longitudinal follow‐ups to evaluate the durability of psychedelic retreat effects, and the potential need for additional retreats to maintain benefits. Clinical integration frameworks are also crucial in sustaining benefits over time, however, there is currently no standardized approach to integration support (Thal et al. 2023). Further studies should aim to compare alternative integration frameworks (e.g., CBT vs. mindfulness‐based models) and frequency of integration sessions to determine which approach best supports long‐term therapeutic outcomes of psychedelic retreat programs in veterans.

Given the prevalence of head trauma and blast force exposure in military populations (Phipps et al. 2020), future research should place greater emphasis on cohorts with definitively reported head injuries. For example, by including detailed cognitive function and neurological health assessments before and after the retreat. The inclusion of head trauma measures would provide deeper insights into the potential effects of psychedelics on physical, as well as psychological, trauma. Particularly given preliminary findings that psychedelics could aid patients’ recovery from physical brain injuries, such as TBI, by modulating neuroplasticity, neuroinflammation, and brain complexity (Khan et al. 2021; Allen et al. 2024).

## 5 Conclusions

This is the first study to explore the effects of a psychedelic retreat program on military veterans and their reintegration into civilian life, revealing significant improvements across all outcome measures related to general and psychological well‐being four weeks after attending a psychedelic retreat. Participants with a reported PTSD diagnosis did not show greater improvements than the general cohort, which could suggest a broader mechanism of action underlying therapeutic benefits rather than psychedelics specifically targeting PTSD pathology.

The study revealed significant improvements in veterans’ reintegration into civilian life following a psychedelic retreat program. Future research should attempt to separate the beneficial impacts of psychedelic medicines’ pharmacological and psychological effects from the supportive, communal environment inherent in the retreat setting. Nonetheless, these improvements highlight the potential of psychedelic retreat programs as a valuable tool to support the complex process of veterans’ reintegration into civilian life through a multi‐disciplinary therapeutic framework.

Psilocybin and ayahuasca may offer distinct benefits depending on the specific challenges experienced by veterans. While psilocybin retreats were associated with greater reductions in most measures, ayahuasca retreats led to slightly greater improvements in PTSD symptoms. These findings suggest that tailoring psychedelic retreats based on individual symptom profiles—for example, psilocybin for post‐deployment reintegration and ayahuasca for PTSD—could enhance therapeutic outcomes.

Lastly, participants who entered the retreat with more severe symptoms of depression and PTSD—indicated by higher baseline scores— experienced more substantial percentage improvements in these symptoms following the retreat. This indicates that psychedelic retreats may offer significant benefits for veterans with more severe symptoms.

Together, these results add support that psychedelic retreats can offer a therapeutic paradigm to treat psychological distress experienced by veterans and the complex transition into civilian life.

## Author Contributions

**Megan Calnan**: data curation, formal analysis, writing – original draft, writing – review and editing. **Grace Blest‐Hopley**: methodology, investigation, writing – original draft, writing – review and editing, supervision. **Chris Busch**: formal analysis, visualization. **Milly Adams**: writing – original draft, writing – review and editing. **Simon G. D. Ruffell**: methodology, investigation. **Theodore Piper**: investigation. **Leor Roseman**: conceptualization. **Hannes Kettner**: methodology, investigation. **Robin Carhart‐Harris**: conceptualization.

## Conflicts of Interest

Beckley retreats provided support in the form of resources but did not influence the study design, data collection, analysis, or interpretation of results. Robin Carhart‐Harris reported receiving consulting fees from COMPASS Pathways, Entheon Biomedical, Medicine, Synthesis Institute, Tryp Therapeutics, and Usona Institute. The other authors declare that the research was conducted in the absence of any commercial or financial relationships that could be construed as a potential conflict of interest.

## Peer Review

The peer review history for this article is available at https://publons.com/publon/10.1002/brb3.70660

## Supporting information



Supplementary Material

## Data Availability

The datasets generated and analysed during the current study are not publicly available due to privacy concerns but are available from the corresponding author on reasonable request. Data will be shared in accordance with relevant guidelines and regulations, ensuring the anonymity and confidentiality of the participants.
