# Supplementary material for: Exploring the Therapeutic Effects of Psychedelics Administered to Military Veterans in Naturalistic Retreat Settings
Source: Brain Behav. 2025 Jul 7;15(7):e70660. doi: 10.1002/brb3.70660 (PMC12230355; doi:10.1002/brb3.70660)
Supplement: Supplementary file 1 — Supplementary Material [file BRB3-15-e70660-s001.docx]

Supplementary Material – Exploring the Therapeutic Effects of Psychedelics Administered to Military Veterans in Naturalistic Retreat Settings

**Figures S1-8.** Change in Questionnaire Scores Pre- and Post- Retreat

1
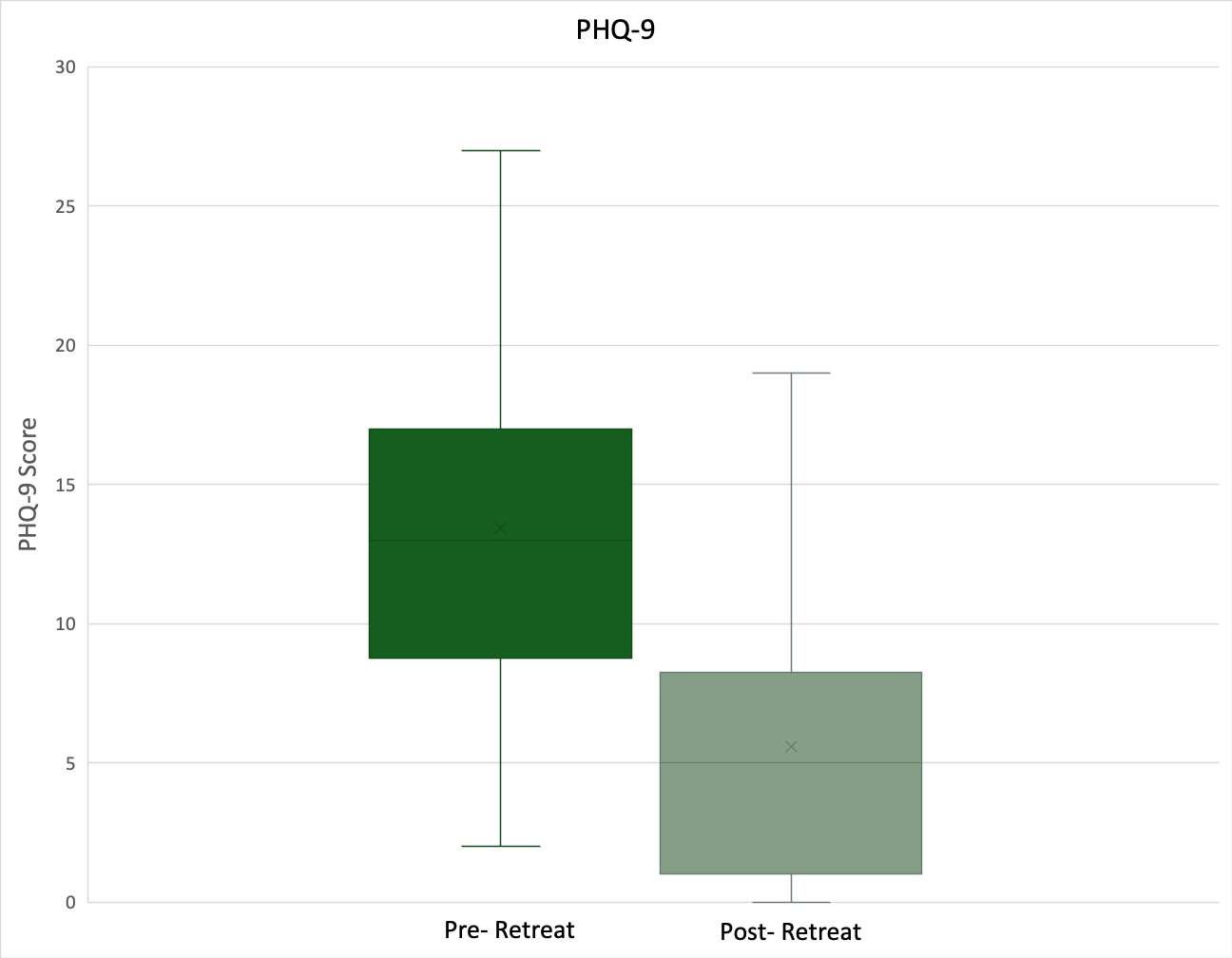


2
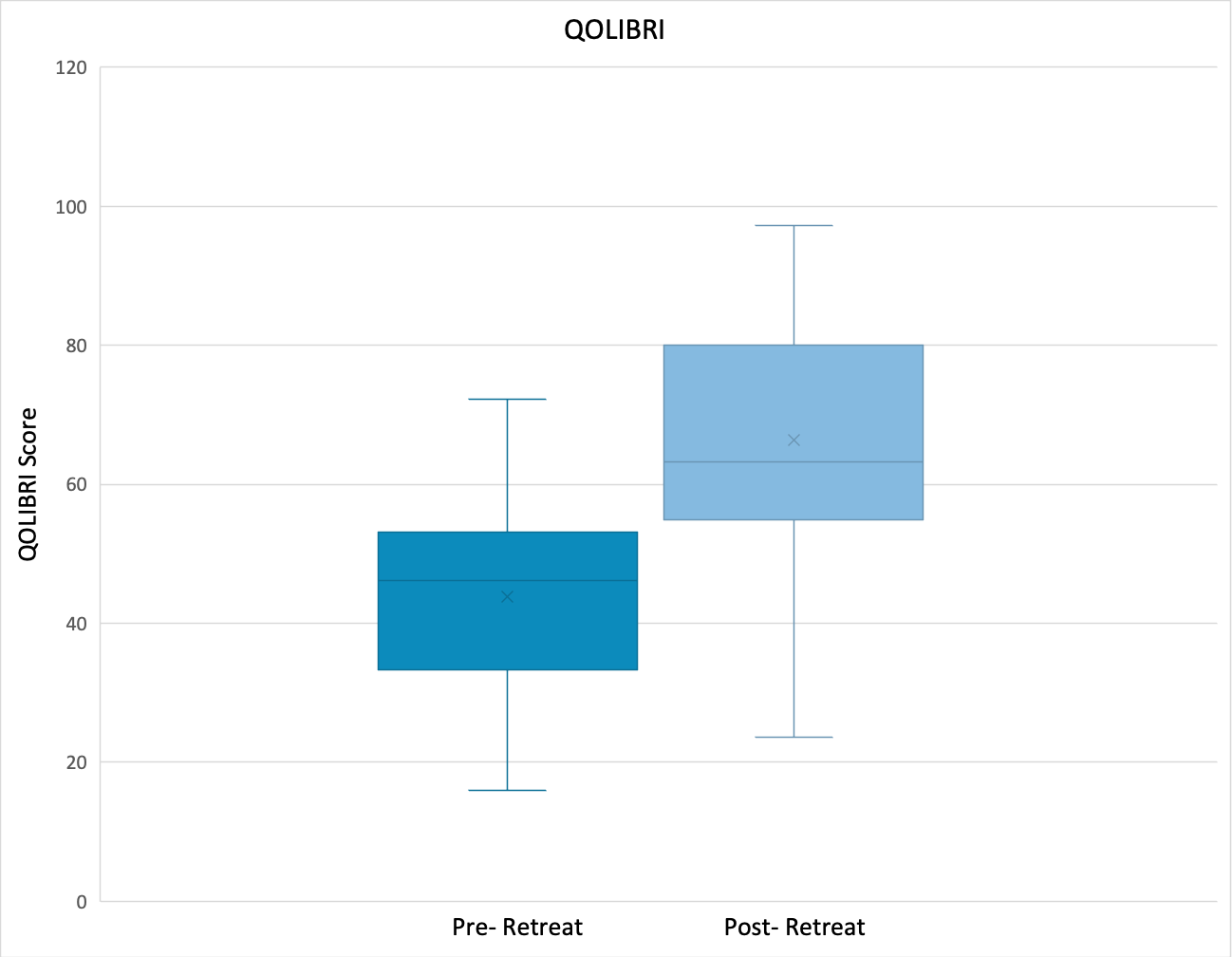


3


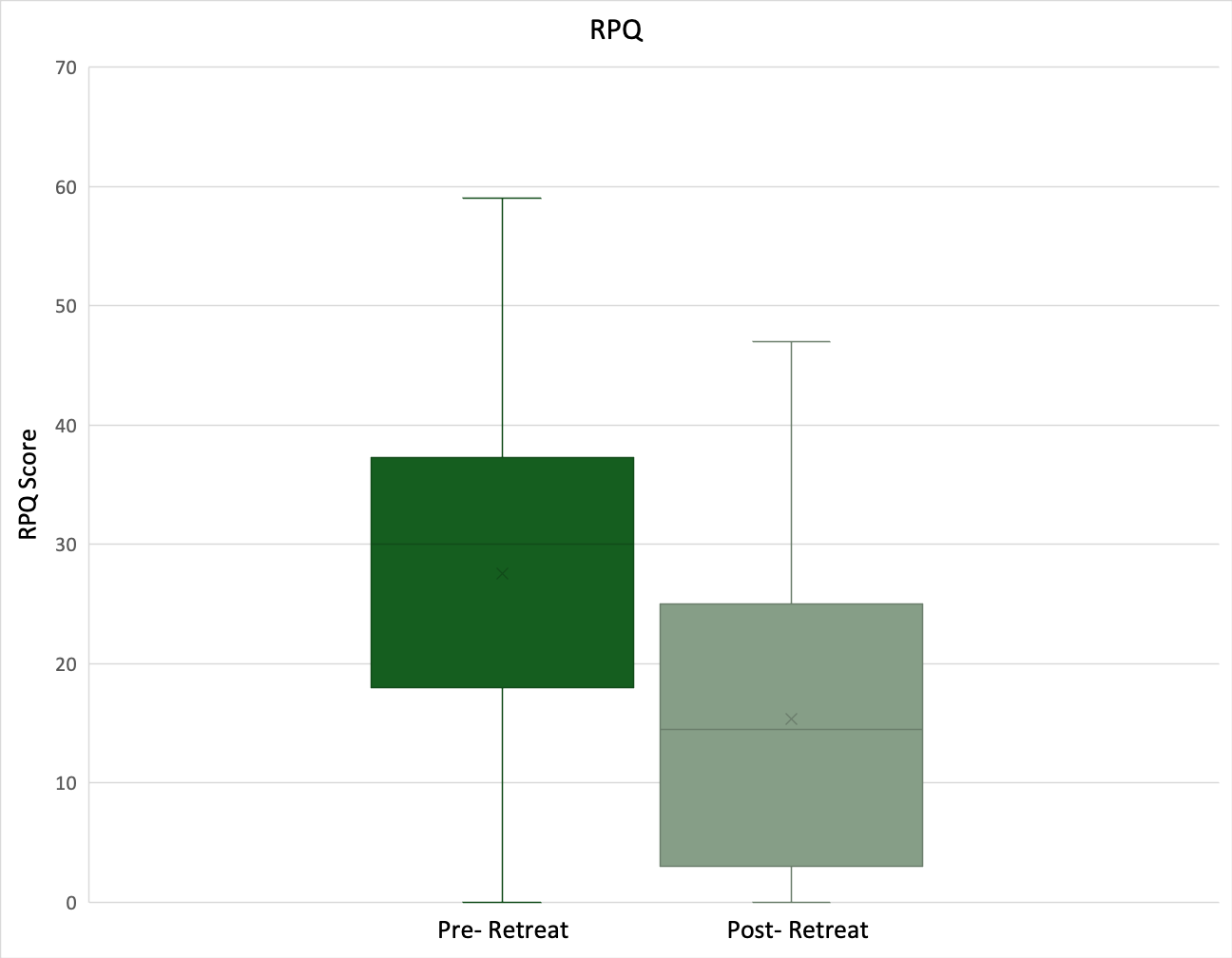


4


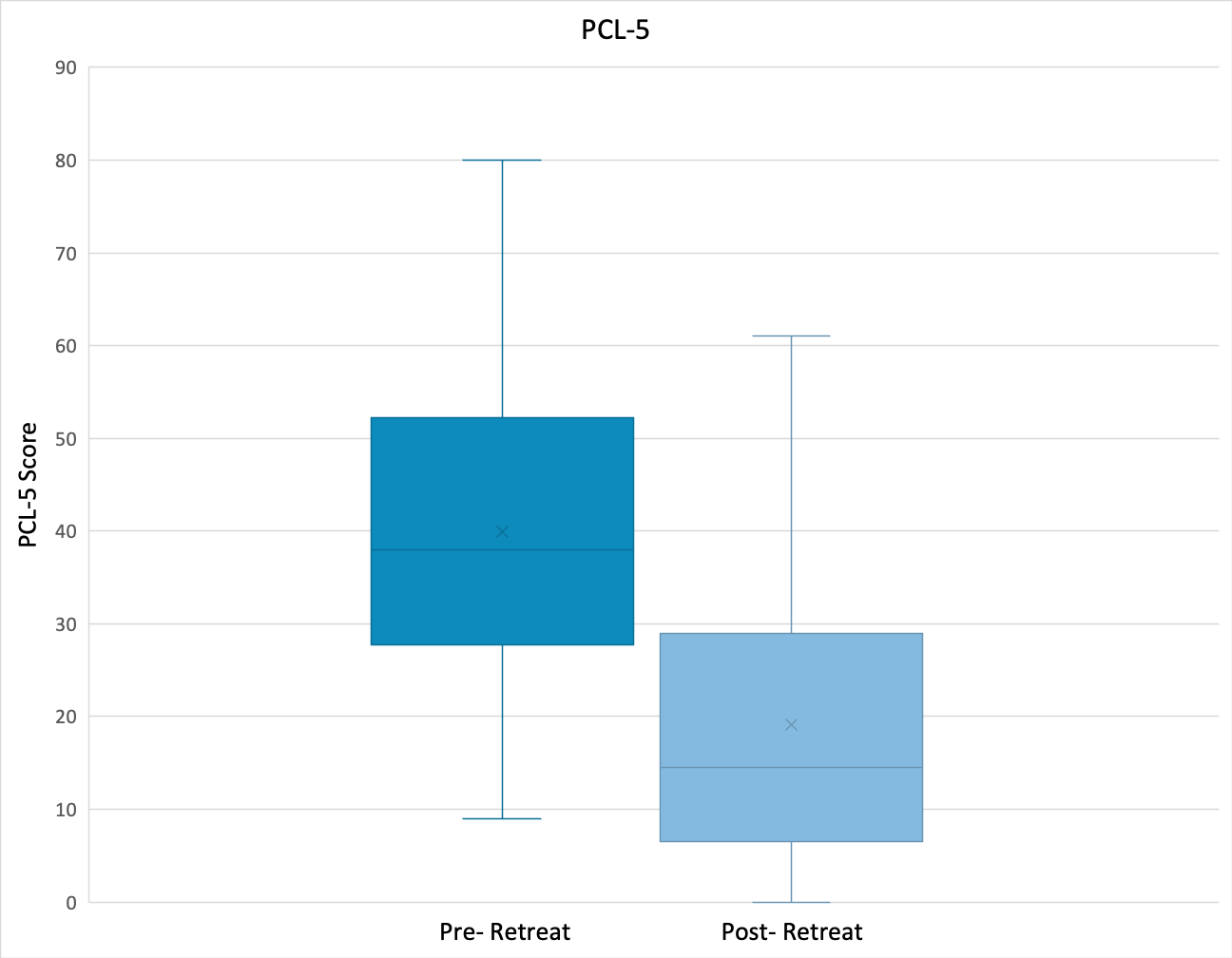


5


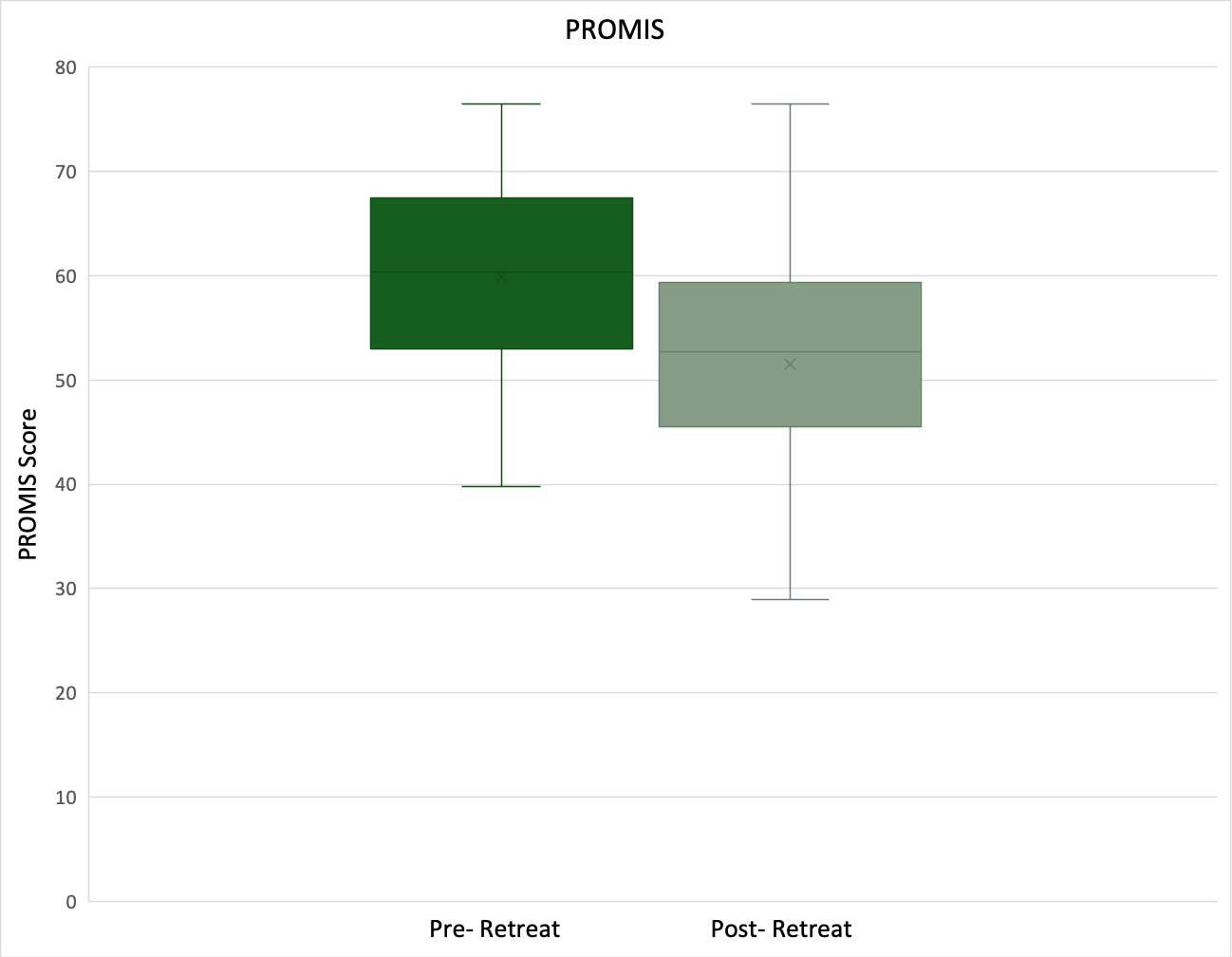


6


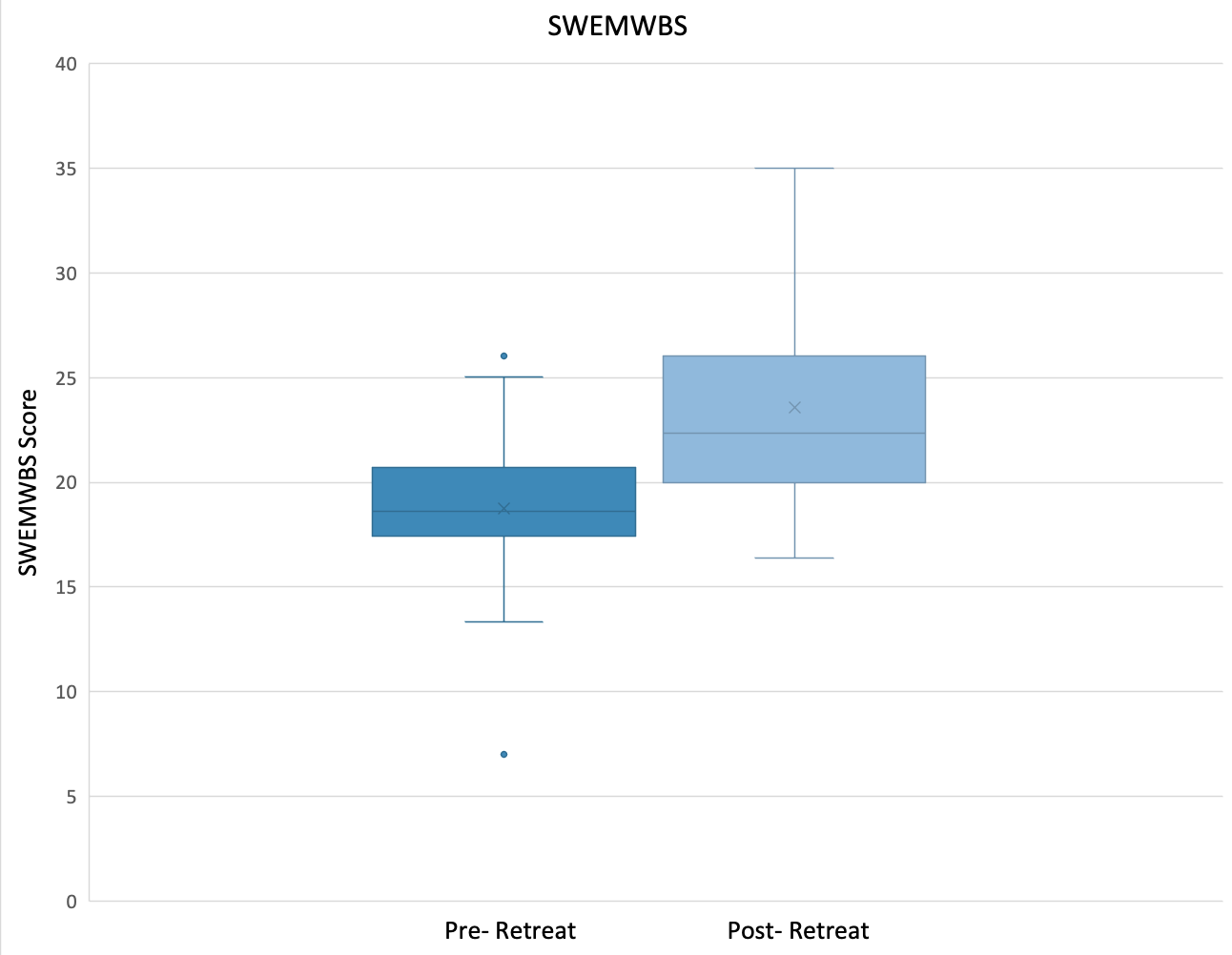


7
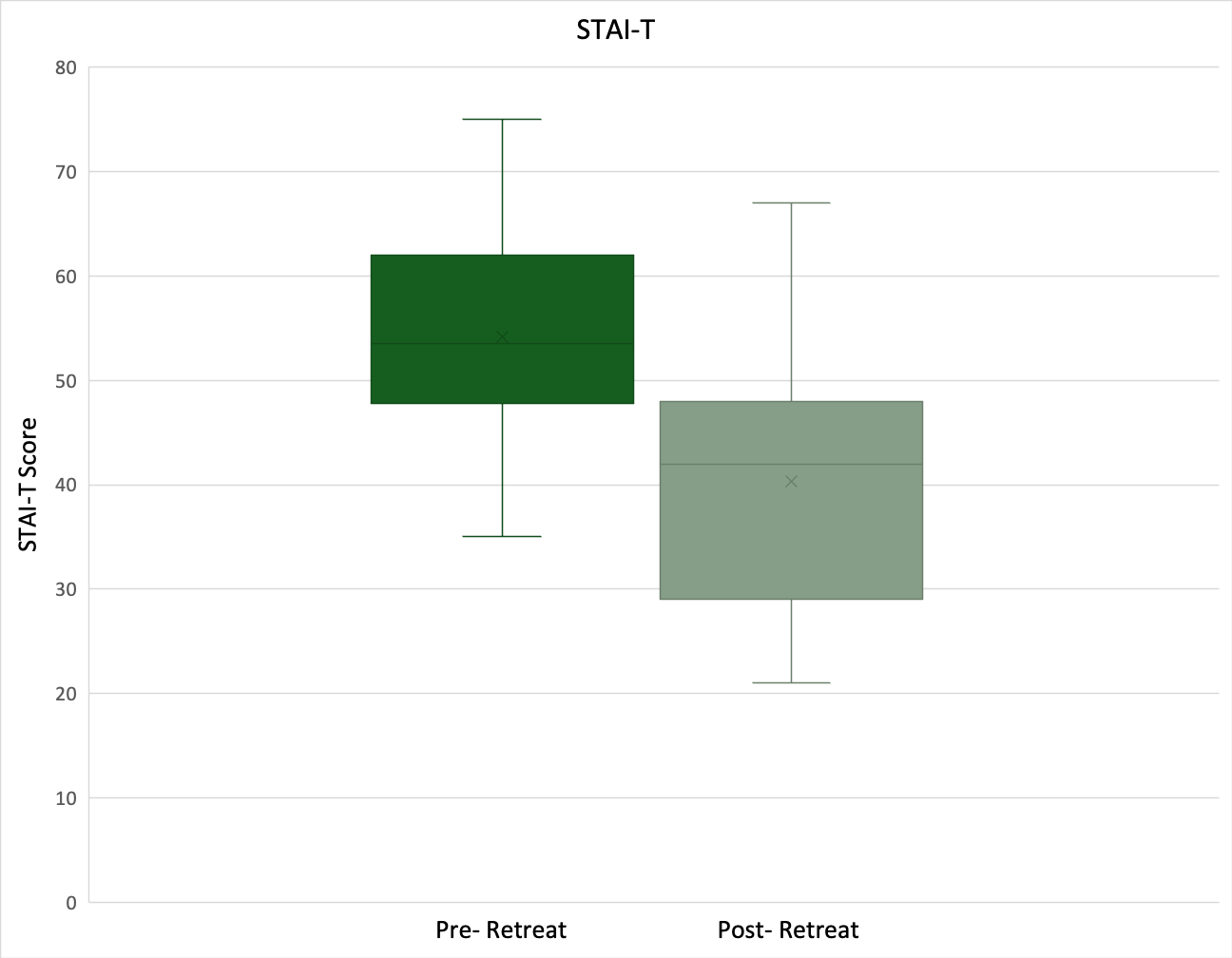


8


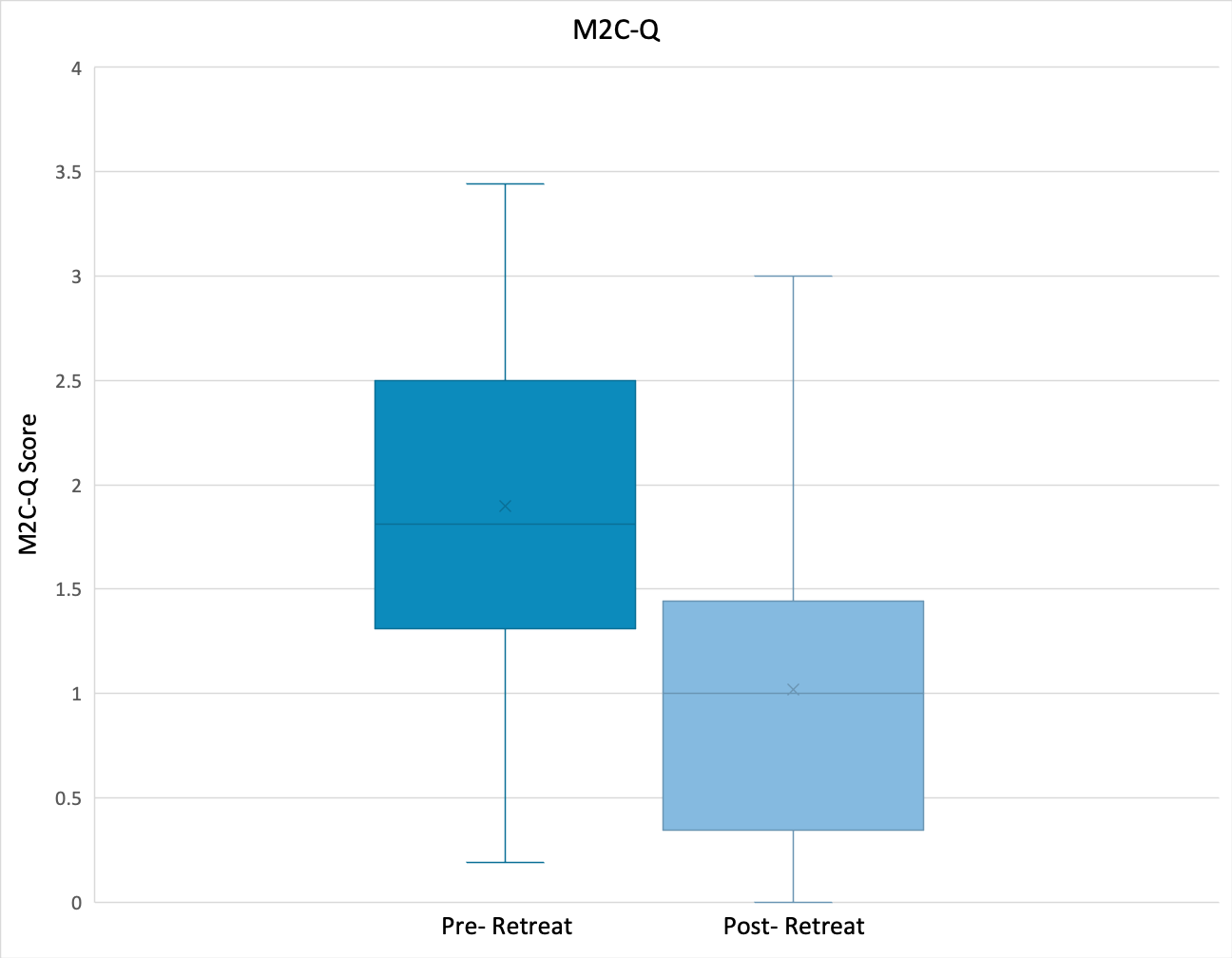


**Figure S9** Distribution of percentage improvement for all participants (N=58) across all outcome measures


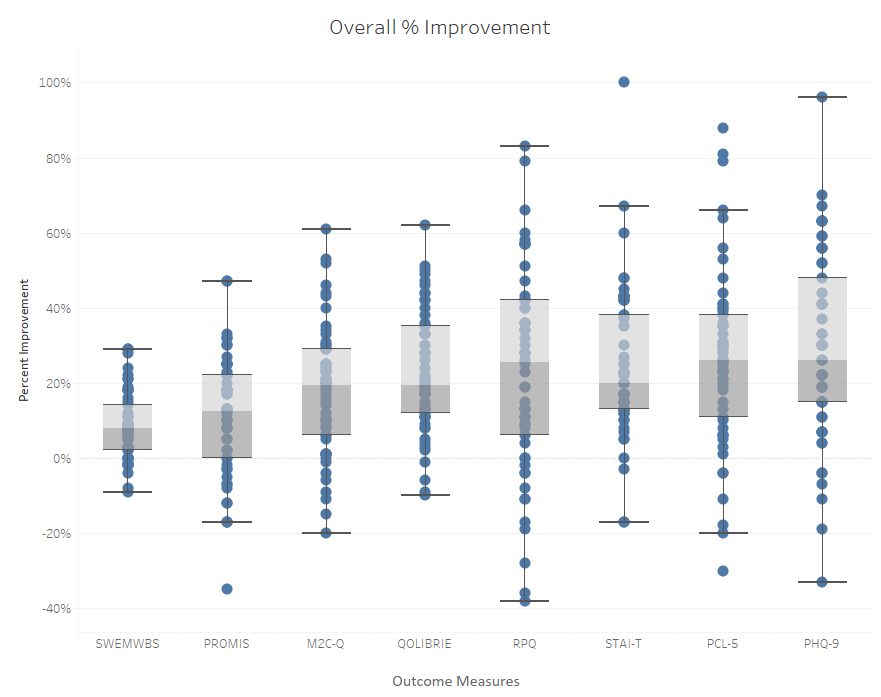
M2C-Q: Military to Civilian Questionnaire; PHQ-9: Patient Health Questionnaire; PLC-5: PTSD Checklist; PROMIS: Patient-Reported Outcomes Measurement Information System; QOLIBRI: Quality of Life After Brain Injury; RPQ: Rivermead Post-Concussion Symptoms; STAI-T: State-Trait Anxiety Inventory; SWEMWBS: Short Warwick-Edinburgh Mental Wellbeing Scales.
